# The state of pediatric asthma in Chicago's Humboldt Park: a community-based study in two local elementary schools

**DOI:** 10.1186/1471-2431-10-45

**Published:** 2010-06-24

**Authors:** Ruchi S Gupta, Juana Ballesteros, Elizabeth E Springston, Bridget Smith, Molly Martin, Eileen Wang, Maureen Damitz

**Affiliations:** 1 Institute for Healthcare Studies, Northwestern University Feinberg School of Medicine; 750 N Lake Shore Dr, 10th Fl, Chicago, IL, 60611, USA; 2 Smith Child Health Research Program, Children's Memorial Hospital; 2300 Children's Plaza, Box 157, Chicago, IL, 60614, USA; 3 The Greater Humboldt Park Community of Wellness; 1116 North Kedzie St, Chicago, IL, 60651, USA; 4 Center for Management of Complex Chronic Care, Edward Hines Jr. VA Hospital; 5000 South 5th Ave, Hines, IL, 60141, USA; 5 Health Services Research Program, Loyola University Stritch School of Medicine; 2160 South 1st Ave, Maywood, IL USA 60153; 6 Departments of Preventive Medicine and Pediatrics, Rush University Medical Center; 1700 W Van Buren St, Suite 470, Chicago, IL, 60612, USA; 7 University of Michigan Medical School; 1301 Catherine Rd, Ann Arbor, MI, 48109, USA; 8 Respiratory Health Association of Metropolitan Chicago; 1440 W Washingston Blvd, Chicago, IL, 60607, USA

## Abstract

**Background:**

Pediatric asthma is a serious public health problem in Chicago and has been designated a high priority concern by residents of Chicago's Humboldt Park, a diverse community area with a large number of Puerto Rican, African American, and Mexican American families.

**Methods:**

In May 2009, following the principles of community-based participatory research, a cross-sectional asthma screening survey was administered to adult caregivers of children attending two Humboldt Park elementary schools. Data were analyzed to determine the prevalence of diagnosed and probable asthma as well as the degree of asthma control among affected children; associations between asthma outcomes and mutable triggers were evaluated.

**Results:**

Surveys from 494 children were evaluated. Physician-diagnosed asthma was reported for 24.9% of children and probable asthma identified in an additional 16.2% of children. Asthma was poorly or moderately controlled in 60.0% of diagnosed children. Smoking occurred inside 25.0% of households and 75.0% of caregivers reported idling of vehicles in their community. Report of general stress among caregivers, stress due to community crime, and/or an inability to cope with everyday life were significantly and positively associated with poor asthma morbidity and control among affected children.

**Conclusions:**

Despite high prevalence rates and poor asthma morbidity and control in Humboldt Park, the association of these measures with mutable variables is promising. A community-based asthma intervention to address the issues identified in this study is needed to affect positive change.

## Background

Childhood asthma is a serious and growing health concern, with prevalence rates at a historic high and over 9 million children in the U.S. diagnosed in their lifetime [1,2]. A costly[3] and potentially debilitating disease, asthma is the most common cause of childhood disability and results in school absenteeism, restricted activity, and increased use of physician and hospital services [4]. Despite improvements in care and increased public awareness, racial and ethnic health disparities persist [2,5]. Minority populations in the U.S. have been shown to have disproportionately higher rates of asthma prevalence, adverse health outcomes and disability [2,4].

In Chicago, asthma has been documented as a serious pediatric health concern, with asthma prevalence, hospitalization, and mortality rates well above the national average [6,7]. A recent study on the geographic variability of childhood asthma found prevalence rates ranging from 2% to 44% by neighborhood [8]. While neighborhood racial composition explained a large proportion of observed variability, the study concluded further research into neighborhood characteristics was needed to better understand persistent disparities. Several social and community factors -- such as exposure to violence,[9-11] ambient air pollution,[12-14] environmental tobacco smoke,[13,14] indoor allergen levels,[15] and caregiver stress[16] -- have been associated with childhood asthma prevalence and morbidity, and may contribute to disparate asthma outcomes.

The purpose of this study was to characterize childhood asthma in Chicago's Humboldt Park neighborhood, a diverse community with a large percentage of Puerto Rican, African American, and Mexican American families. In 2005, community organizers designated asthma a high priority concern and sought partners in academia to address the problem. Multiple initiatives resulted, including the community-academic partnership described herein.

## Methods

The design of this research project utilized community-based participatory research (CBPR), a collaborative approach to research that addresses a topic of importance to the community and equitably involves academic and community partners in the conduct of research. A CBPR partnership between Children's Memorial Hospital, the Greater Humboldt Park Community of Wellness, the Respiratory Health Association of Metropolitan Chicago, and Rush University Medical Center was formed to evaluate asthma in Humboldt Park by surveying adult caregivers of children attending two public elementary schools.

The Institutional Review Boards of Children's Memorial and Rush University as well as the Chicago Public Schools Research Review Board approved the study protocol. Administration of the survey was conducted with consent of participating schools. Caregiver consent to participate was implicit in the return of completed surveys, which did not include personal identifiers.

### Study Sample

Caregivers of children attending Von Humboldt and Ana Roque de Duprey Elementary schools in Humboldt Park were sought to participate in a cross-sectional survey to evaluate community asthma. Schools were chosen to be representative of the broader demographic characteristics of Humboldt Park (Table 1). Adult caregivers (≥18 yrs) able to complete a written survey in Spanish or English were eligible and asked to participate. Non-participation was due to inability to complete the survey in Spanish or English, child's absence from the classroom during survey distribution, or failure to return the survey.

**Table 1:** Demographic characteristics of Humboldt Park and targeted Humboldt Park elementary

Characteristic	Humboldt Park	Von Humboldt Elementary	Ana Roque de Duprey Elementary
Population size (n)	65,836	624	296
% Hispanic	48.0	52.4	80.5
% Puerto Rican	37.3	--	--
% Black	47.4	41.0	10.8
% White	3.3	0.6	3.0
% Asian	0.4	0.0	0.9
% Other race/ethnicity	0.9	6.0	4.8
% Low income^i^	88.6	97.9	97.0
% Limited English proficiency^ii^	31.2	4.5	33.3

^i^Low-income in Humboldt Park defined as the percentage of residents at or below the poverty level; low-income in elementary schools defined as the percentage of students receiving free/reduced lunch

^ii^Limited English proficiency defined as report of speaking English "not well" or "not at all"

Sources: U.S. Census Bureau, Census 2000. Chicago Public School Profiles, Data as of 2008, available at <https://research.cps.k12.il.us/resweb/PageServlet?page=schoolprofile&class=profile.SchoolProfile>.

### Survey Instrument

The survey instrument was developed using well-established survey methodology [17,18]. Initial survey items were constructed by the research team, which included pediatricians, survey researchers, experts in respiratory health, and community leaders. The validity of the instrument was assessed through cognitive interviews with community members to ensure understandability (n = 10, six English/four Spanish). Items were then subject to reliability testing to account for the consistency of participant response over time (n = 10, five English/five Spanish). The final survey included 35 items and consisted of questions regarding the child's demographic characteristics, details on home environment, parental report of stress and exposure to violence, parental evaluation of community, assessment of probable asthma,[19] parental report of physician/nurse diagnosed asthma, assessment of asthma control,[20] report of medication usage, and perception of asthma management in the schools.

### Study Protocol

Parents and school staff were informed of the study in a letter from the school principal and community partners. Surveys were distributed in three waves to 36 pre-kindergarten to 8^th ^grade classrooms during May of 2009. Children were instructed to take the survey home to be completed by an adult caregiver. Completed surveys were held by classroom teachers and returned to investigators on a weekly basis. Teachers were instructed to mark students who had and had not returned the survey on their attendance list; physical surveys contained no personal identifying information. Additional surveys were distributed each week to students whose caregivers had not yet responded. Students were given a pencil and eraser when they returned the survey. Classrooms achieving a response rate above 70% received a pizza party. All teachers were given a $5 gift card in appreciation for their time.

### Analysis

Response frequencies were calculated to examine the prevalence of diagnosed and probable asthma. The frequency of reported medication use and response to survey items addressing asthma morbidity and control were calculated for children with a reported diagnosis of asthma. Demographic characteristics and triggers were stratified by the child's asthma status. Finally, to examine the association between asthma outcomes and potential triggers, prevalence ratios (PR) were estimated using Poisson regression models with robust error variances [21].

Respondents who did not indicate whether or not their child had a formal diagnosis of asthma were excluded from analysis (n = 22). Returned surveys with missing responses to other items were included. Accordingly, sample size for each item varied as noted in the tables.

All statistical analyses were performed using STATA 10 (StataCorp LP; College Station, TX) with a Type I error of p < 0.05.

## Results

Surveys were distributed to 874 students and returned for 494 children (56.5%, Table 2). Children primarily fell between the ages of 7 to 14 years and most reported being Puerto Rican (41.4%), Black (33.5%), and/or Mexican (32.5%). The majority of children received public assistance in the form of Medicaid/AllKids (82.4%).

**Table 2:** Demographic characteristics of children surveyed.^i^

Variable	Proportion (%)
Asthma status	
Diagnosed asthma (n = 494)	24.9
Probable asthma^ii ^(n = 488)	16.2
Age (years, n = 491)	
3-4	4.7
5-6	7.7
7-8	21.6
9-10	21.2
11-12	18.8
13-14	22.4
15-16	3.5
Race/ethnicity (n = 494)^iii^	
Black	33.5
White	7.7
Asian	0.2
Puerto Rican	41.4
Mexican	32.5
Other Hispanic	11.8
Other ethnicity	1.2
Insurance type (n = 482)	
Private	8.7
Medicaid/AllKids	82.4
Uninsured	7.7
Other	1.2

^i^Response rate varied by item; n-value listed indicates the total number of responses received for a given item.

^ii^Probable asthma defined as 1 or more affirmative response to the Brief Pediatric Asthma Screen Plus[19] without having a formal diagnosis of asthma.

^iii^Multiple selections were allowed; sum need not equal 100%.

### Asthma Prevalence, Morbidity, and Control

One in four caregivers (24.9%) reported having a child with physician-diagnosed asthma (Table 2). Probable asthma, defined as at least one affirmative response to the Brief Pediatric Screen Plus,[19] was identified in 16.2% of children without a formal asthma diagnosis. Among children diagnosed with asthma, 48.0% reported using long-term asthma control medication and 68.3% quick-relief medication during the month prior to the survey (Table 3); 81.5% of caregiver's believed their child's medication helped his or her asthma. Asthma was moderately or poorly controlled among 60.0% of children based on an affirmative response to one or more questions on items 1a-e of the Asthma Therapy Assessment Questionnaire [20]. Roughly 1 in 3 asthmatic children had missed school in the past month and had visited a doctor or the emergency department in the past year due to their illness; 5.4% of children were admitted to the hospital for an asthma exacerbation in the year prior to the survey.

**Table 3:** Asthma morbidity and control among children with a reported diagnosis of asthma.^i^

Variable	Frequency (%)
Reported use of long-term asthma control medication (past month, n = 123)^ii^	
Inhaled corticosteroids^iii^	28.5
Long-acting beta-2 agonists^iv^	0.8
Leukotriene modifiers^v^	13.0
Cromolyn, nedocromil^vi^	0
Combination medication^vii^	5.7
Reported use of quick-relief medication (past month, n = 123)^ii^	
Short-acting beta-2 agonists^viii^	68.3
Asthma control (n = 100)^ix^	
Poorly controlled	27.00
Moderately controlled	33.00
Well controlled	40.00
Child missed school because of asthma? (past month, n = 104)	
Yes	31.7
No	68.3
Child visited doctor/ED for asthma? (past year, n = 103)	
Yes	35.9
No	64.1
Child admitted to hospital for asthma? (past year, n = 104)	
Yes	5.8
No	94.2
Believe medications help child's asthma (n = 103)?	
Yes	81.5
No	7.8
Unsure	10.7
Comfortable school can manage child's asthma (n = 105)?	
Not at all	11.4
Somewhat	41.0
Very	47.6

^i^Response rate varied by item; n-value listed indicates the total number of responses received for a given item.

^ii ^Multiple selections were allowed; sum need not equal 100%.

^iii^Pulmicort Flexhaler, Azmacort, Qvar (40 and 80 mcg), Pulmicort Respules, Prednisone/Prednisolone, Flovent

^iv^Serevent DPI

^v^Singulair

^vi^Intal

^vii^Advair, Advair MDI

^viii^Albuterol, Proventil, Ventolin, Pro Air, Xopenex, Maxair, Nebulizer

^ix^Levels of asthma control defined by responses to items 1a-e on the Asthma Therapy Assessment Questionnaire:[20] Poorly controlled → 3 or more affirmative responses; Moderately controlled → 1-2 affirmative responses; Well controlled → 0 affirmative responses

### Frequency of Individual, Community, and Psychosocial Triggers

Triggers associated with childhood asthma were evaluated for all participants on individual, community, and psychosocial levels. Children tended to live in homes rented by their caregiver (83.6%). Inside the home, 25.0% of children were exposed to environmental tobacco smoke and 11.9% of caregivers observed roaches. Indoor pets were frequently reported, with 21.1% of children having dogs and 14.0% having cats.

Half of all participants (51.2%) reported living in Humboldt Park for five years or more. Caregivers regularly observed vehicles idling on the streets, with 75.0% reporting idling at least some of the time. Safety in the neighborhood was also a concern, with 63.0% of caregivers feeling unsafe at least some of the time and the majority (57.4%) keeping children inside because of violence.

Stress among caregivers was common, with 68.4% generally stressed and 51.4% stressed by community crime at least some of the time in the month prior to completing the survey. In the same time period, 48.4% of caregivers felt that they were unable to cope with their responsibilities at least some of the time. Frequencies for all triggers were either similar or elevated for children with asthma and probable asthma (Table 4).

**Table 4:** Characteristics of children surveyed presented by asthma status.^i^

Variable	Proportion (%), Diagnosed Asthma	Proportion (%), Probable Asthma^ii^	Proportion (%), No Asthma
**Demographic characteristics**			
Age (years)	(n = 122)	(n = 78)	(n = 287)
3-4	4.9	5.1	4.2
5-6	5.7	11.5	7.3
7-8	18.0	24.4	22.3
9-10	23.0	21.8	20.2
11-12	19.7	14.1	20.2
13-14	26.2	18.0	22.3
15-16	2.5	5.1	3.5
Race/Ethnicity^iii^	(n = 123)	(n = 79)	(n = 288)
Black	43.1	34.2	29.6
White	6.5	2.5	6.3
Asian	0.0	0.0	0.4
Puerto Rican	49.6	29.1	36.7
Mexican	21.1	17.7	37.5
Other Hispanic	12.2	7.6	12.9
Other Ethnicity	1.6	0.0	1.4
Insurance Type	(n = 121)	(n = 73)	(n = 284)
Private	8.3	11.0	8.8
Medicaid/AllKids	81.8	78.1	83.5
Uninsured	8.3	9.6	6.7
Other	1.7	1.4	1.1
**Individual triggers**			
Child lives in:^iii^	(n = 105)	(n = 74)	(n = 221)
Rented home	85.7	77.0	84.6
Live with family/friends	8.6	9.5	7.7
Owned home	8.6	16.2	10.0
Smoking inside child's home?	(n = 105)	(n = 74)	(n = 220)
Yes	23.8	29.7	24.5
No	76.2	68.9	75.0
Unsure	0.0	1.4	0.5
Roach inside child's home?	(n = 105)	(n = 74)	(n = 220)
Yes	12.4	13.5	10.9
No	84.8	78.4	88.6
Unsure	2.9	8.1	0.5
Pet inside child's home?^iii^	(n = 104)	(n = 72)	(n = 220)
Yes, cat	15.4	15.3	12.7
Yes, dog	24.0	19.4	20.0
Yes, other pet	11.5	9.7	7.3
No	54.8	61.1	60.0
**Community triggers**			
Time in neighborhood?	(n = 122)	(n = 79)	(n = 286)
≥ 5 years	54.1	51.9	47.9
< 5 years	45.9	48.1	52.1
Vehicles idle on street?	(n = 119)	(n = 78)	(n = 281)
Most of the time	25.2	31.7	16.0
Some of the time	51.3	48.1	57.7
None of the time	23.5	20.3	26.3
Felt unsafe in neighborhood?	(n = 123)	(n = 79)	(n = 79)
Most of the time	16.3	31.7	10.6
Some of the time	53.7	50.6	46.8
None of the time	30.1	17.7	42.6
Ever kept child inside because of violence in community?	(n = 123)	(n = 75)	(n = 219)
Yes	64.1	61.3	52.5
No	35.9	38.7	47.5
**Psychosocial triggers**			
Felt nervous/stressed? (past month)	(n = 123)	(n = 75)	(n = 285)
Most of the time	15.5	11.4	13.3
Some of the time	60.2	59.5	50.9
None of the time	24.4	29.1	35.8
Nervous/stressed by crime in community? (past month)	(n = 123)	(n = 79)	(n = 285)
Most of the time	11.5	20.3	11.2
Some of the time	44.3	31.7	37.6
None of the time	44.3	48.1	51.2
Could not cope with responsibilities? (past month)	(n = 102)	(n = 73)	(n = 220)
Most of the time	6.9	5.5	4.6
Some of the time	47.1	50.7	56.8
None of the time	46.1	43.8	38.6

^i^Response rate varied by item; n-value listed indicates the total number of responses received for a given item.

^ii^Probable asthma defined as 1 or more affirmative response to the Brief Pediatric Asthma Screen Plus[19] without having a formal diagnosis of asthma.

^iii^Multiple selections were allowed; sum need not equal 100%.

### Associations

The associations between asthma outcomes and the triggers investigated were estimated using prevalence ratios (PR) obtained from multiple Poisson regression models (Table 5).

**Table 5:** Variables significantly associated with asthma, probable asthma,^i ^and asthma morbidity/control.^ii^

Variable	Prevalence Ratio (PR)	95% Confidence Interval (CI)
**Risk of asthma diagnosis**		
Black vs. all other races/ethnicities	1.51	1.11-2.04
Puerto Rican vs. all other races/ethnicities	1.39	1.03-1.89
Mexican vs. all other races/ethnicities	0.56	0.38-0.82
**Risk of probable asthma**		
High vs. low vehicle idling	1.80	1.18-2.74
Caregiver stressed by community crime	1.73	1.07-2.79
**Risk of wheeze during the day (diagnosed asthma only)**		
Caregiver felt unable to cope	2.39	1.17-4.88
Caregiver stressed by community crime	2.07	1.05-4.88
**Risk of wheeze at night (diagnosed asthma only)**		
Caregiver felt unable to cope	2.24	1.09-4.58
**Risk of wheeze during exercise (diagnosed asthma only)**		
Caregiver felt unable to cope	1.68	1.03-2.74
Caregiver reported general stress	2.05	1.03-4.07
**Risk of missed school days (diagnosed asthma only)**		
Caregiver felt unable to cope	2.45	1.21-4.96

^i^Probable asthma defined as 1 or more affirmative response to the Brief Pediatric Asthma Screen Plus[19] without having a formal diagnosis of asthma.

^ii^Prevalence ratios for all variables listed in Table 4 were estimated; only statistically significant associations are shown here.

#### Risk of asthma

Risk of being diagnosed with asthma was significantly higher among children of Black or Puerto Rican race/ethnicity (PR = 1.51, 95% CI 1.11-2.04 and PR = 1.39, 95% CI 1.03-1.89 respectively) and significantly lower among Mexican children (PR = 0.56, 95% CI 0.38-0.82) when compared to all other races/ethnicities. A significantly elevated risk of probable asthma was observed only for children whose caregivers observed the highest levels of vehicle idling or experienced stress due to crime most frequently in the month prior to the survey (report of most of the time vs. some/none of the time: PR = 1.80, 95% CI 1.18-2.74 and PR = 1.73, 95% CI 1.07-2.79 respectively). Increased risk was not observed among caregivers reporting idling or stress due to crime at lower levels.

#### Risk of poor morbidity and control in children with asthma

Among children diagnosed with asthma, those whose caregivers felt unable to cope were more likely to wheeze during the day (PR = 2.39, 95% CI 1.17-4.88), wheeze while exercising (PR = 1.68 95% CI 1.03-2.74), wheeze at night (PR = 2.24, 95% CI 1.09-4.58) and to miss school due to asthma (PR = 2.45, 95% CI 1.21-4.96). Wheezing during the day was also more likely among children whose caregivers reported stress due to crime (PR = 2.07, 95% CI 1.05-4.88). Risk of wheeze during exercise was higher for children whose caregivers experienced general stress (PR = 2.05, 95% CI 1.03-4.07).

## Discussion

Pediatric asthma prevalence in Humboldt Park was exceptionally high among children in our study. At 25%, it was more than twice the national average [2]. Also troubling, probable asthma was identified in 17% of children without a formal diagnosis of asthma. As of 2004, asthma in Humboldt Park was estimated to affect 17% of children, with probable asthma reported in an additional 11% of children [22]. Our findings suggest that pediatric asthma in this community remains a pressing concern, and, indeed, may be on the rise. Consistent with previous estimates of racial/ethnic asthma disparities in Chicago,[6,22,23] asthma was significantly more likely among Black and Puerto Rican children compared to all other races/ethnicities. Such findings point once more to the tremendous asthma burden born by minority families in urban environments.

Smoking in households with and without asthmatic children was reported frequently, though not as often as estimated by other studies in Humboldt Park [22]. Multiple studies suggest that exposure to environmental tobacco smoke significantly increases a child's risk of being diagnosed with asthma and experiencing severe asthma symptomatology [24-26]. Encouragingly, a recent study found that changes in exposure to environmental tobacco smoke were significantly associated with changes in pediatric asthma morbidity; decreased exposure was predictive of fewer asthma-related visits to the emergency department, fewer hospitalizations, and decreased exacerbation odds [27]. However, smoking cessation interventions have had limited success, especially in low-income populations.

Participants in our study, both with and without asthma, frequently reported exposure to other indoor pollutants implicated in asthma outcomes. Specifically, 36% of children had an indoor dog or cat and 12% of caregivers reported cockroaches inside the home. Sensitizations to dog or cat or having a dog in one's home have been identified as risk factors for asthma [24,28]. High levels of cockroach and pet allergen levels in the homes of sensitized children have been associated with increased asthma morbidity and greater asthma severity, respectively [29-31].

Exposure to ambient pollutants from traffic exhaust has also been associated with poor respiratory health. For example, children with the highest exposure to traffic exhaust have been shown to have significantly increased risk of recurrent dry nighttime cough compared to children with lower exposures [32]. A dose-response relationship was also observed in our study, such that only children whose caregivers reported the highest levels of idling were at an increased risk of probable asthma. Notably, reduction in adverse asthma events has been documented in relation to decreased traffic exposures [33].

Emerging literature indicates that stress may play a significant role in pediatric asthma. Research suggests that stress among caregivers is linked to stress response in children [34,35]. Childhood stress, in turn, may predict greater asthma severity and morbidity over time. Among children with asthma, there is evidence that chronic stress coupled with an acute incidence of stress substantially increases the risk for asthma exacerbations [36]. Symptoms of posttraumatic stress have also been associated with more severe asthma in children [37]. Further, parental stress has been prospectively associated with increased risk of childhood wheeze early in life [16]. In line with these findings, a caregiver's inability to cope with daily life, which may indicate a high level of internalized stress, was most strongly and frequently associated with adverse asthma outcomes among affected children in our study. While not to the same extent, caregiver report of general stress was also associated with poor asthma control. Because the stronger association was not report of stress itself, but report of an inability to cope with stress, instruction in parental coping mechanisms may be an important target for intervention in this population.

The impact of violence on childhood asthma is also becoming more evident. Exposure to violence has been significantly associated with poor health outcomes[38] and is often conceptualized as a source of chronic psychological stress [11]. In urban environments, caregivers' self-report of exposure to violence has been significantly associated with more symptom days and nights among children with asthma, as well as with decreased lung function in general [10,39]. Further, the likelihood of asthma has been significantly associated with neighborhood violence, even after controlling for potential confounders [40]. Consistent with these findings, risk of probable asthma and poor asthma morbidity/control in our study was significantly higher among children whose caregivers experienced stress due to community crime. The implication of crime on the community-level illustrates the importance of considering a child's broader social environment when designing interventions to address pediatric asthma.

The impact of neighborhood stability on asthma outcomes, as measured by length of residence and residence type, was unclear in our study. This association is also unclear in the literature. Prior studies have linked more residential stability both with *higher*[41,42] and *lower*[43] asthma rates. Higher asthma rates in more stable communities have been attributed to less thorough and frequent maintenance cleaning in homes occupied for a longer period of time [41]. Conversely, lower asthma rates in more stable communities have been suggested to indicate a better built environment [43].

This study is not without limitations. The cross-sectional study design limits interpretations of causality. The data represent two schools in the Humboldt Park neighborhood with only a moderate response rate and cannot be generalized to the entire community area. However, the sample largely mirrored the demographic characteristics of the schools and of the Humboldt Park community at large, with most respondents from low-income minority homes.  Only adult caregivers able to complete the survey in English or Spanish and receiving the survey from their child were able to participate. Due to an administrative error, data on gender was not collected during survey administration and response rate varied by item. Finally, there may have been some selection bias inherent in the recruitment process; those with a child with asthma may have been more inclined to complete the survey, possibly inflating the measurement of asthma.

## Conclusions

Our study suggests that children in Humboldt Park suffer from tremendously high rates of asthma prevalence and adverse outcomes. However, the positive association of these measures with mutable variables suggests ways to reverse these disparities. Although little can be done to address the genetic components of pediatric asthma at this time, interventions on the community level to combat disease have been efficacious and replicated in multiple urban environments [44]. An intervention designed to specifically address the factors implicated in this study and tailored to meet the needs of Humboldt Park is a warranted and much needed next step to work in this community. Encouragingly, Humboldt Park residents are engaged in their community and committed to improving asthma among their children, both essential components of successful asthma interventions [45]. Our work suggests that community-academic partnerships in activated communities such as Humboldt Park can result in effective research initiatives and hold promise for successful and sustainable asthma interventions.

## Competing interests

The authors declare that they have no competing interests, either real or perceived, in the publication and dissemination of this manuscript.

## Authors' contributions

RG and JB conceived of the study. RG, JB, ES, MM, EW and MD, participated in its design and coordination. RG, JB, ES, and EW oversaw data collection. ES drafted the manuscript, with substantial contributions from RG, JB, BS, MM, EW and MD. BS performed the statistical analysis. All authors read and approved the final manuscript.

## Pre-publication history

The pre-publication history for this paper can be accessed here:

http://www.biomedcentral.com/1471-2431/10/45/prepub
